# Strategic use of new generation antidepressants for depression, SUN(^_^)D : study design and rationale

**DOI:** 10.1186/1745-6215-12-S1-A106

**Published:** 2011-12-13

**Authors:** Naohiro Yonemoto, Tatsuo Akechi, Shinji Shimodera, Mitsuhiko Yamada, Kazuhira Miki, Norio Watanabe, Masatoshi Inagaki, Toshi A Furukawa

**Affiliations:** 1 Department of Epidemiology and Biostatistics, Translational Medical Center, National Center of Mental Health, National Centre of Neurology and Psychiatry, Kodaira, Tokyo 187-8502, Japan; 2 Department of Psychiatry and Cognitive-Behavioral Medicine, Nagoya City University Graduate School of Medical Sciences, Nagoya, Nagoya, 467-8601, Japan; 3 Department of Neuropsychiatry, Kochi Medical School, Nankokushi,Kochi 783-8505, Japan; 4 Department of Neuropsychopharmacology, National Institute of Mental Health, National Center of Neurology and Psychiatry, Kodaira, Tokyo 187-8553, Japan; 5 Miki Clinic, Yokohama, Kanagawa, 220-0023, Japan; 6 Center for Suicide Prevention, National Institute of Mental Health,National Center of Neurology and Psychiatry, Kodaira,Tokyo 187-8553, Japan; 7 Department of Health Promotion and Human Behavior, Kyoto University Graduate School of Medicine/ School of Public Health, Kyoto, Kyoto 606-8501, Japan

## Background

After more than half a century of modern psychopharmacology, with billions of dollars spent on antidepressants annually world-wide, we lack good evidence to guide our everyday decisions in conducting antidepressant treatment of patients with major depression. First we did not know which antidepressant to use as first line treatment. Second we do not know which dosage we should be aiming at with that antidepressant. Because more than half of the patients with major depression starting treatment do not remit after adequate trial with the first agent, they will need a second line treatment. Dose escalation, augmentation and switching are the three often recommended second line strategies but we do not know which is better than the others. Moreover, we do not know when to start considering this second line treatment. The recently published multiple-treatments meta-analysis of 12 new generation antidepressants has provided some partial answers to the first question [1]. Starting with these findings, this proposed trial aims to establish the optimum 1^st^ line and 2^nd^ line antidepressant treatment strategy among adult patients with a non-psychotic unipolar major depressive episode.

## Methods/design

SUN(^_^)D, the Strategic Use of New generation antidepressants for Depression, is an assessor-blinded, parallel-group, multi-centre, pragmatic randomised controlled trial. The trial composes three-steps [2]. Step I is a cluster-randomised trial comparing titration up to the minimum vs maximum of the recommended dose range among patients starting with sertraline. The primary outcome is the change in the Patient Health Questionnaire (PHQ)-9 scores administered by a blinded rater via telephone at week 1 through 3. Step II is an individually randomised trial comparing staying on sertraline, augmentation of sertraline with mirtazapine, and switching to mirtazapine among patients who have not remitted on the first line treatment by week 3.The primary outcome is the change in the PHQ-9 scores at week 4 through 9. Step III represents a continuation phase to Steps I and II and aims to establish longer-term effectiveness and acceptability of the above-examined treatment strategies up to week 25.

## Discussion

The trial is first pragmatic mega trial of psychiatry in Japan. We are now going a pilot phase in the trial. The pilot phase is supported by the Grant-in-Aid by the Ministry of Health, Labour and Welfare, Japan.

Trial registration ClinicalTrials.gov identifier: NCT01109693.
